# Effects of Dual-Channel Functional Electrical Stimulation on Gait Performance in Patients with Hemiparesis

**DOI:** 10.1100/2012/530906

**Published:** 2012-10-11

**Authors:** Shmuel Springer, Jean-Jacques Vatine, Ronit Lipson, Alon Wolf, Yocheved Laufer

**Affiliations:** ^1^Department of Physical Therapy, Faculty of Social Welfare and Health Sciences, University of Haifa, 31905 Haifa, Israel; ^2^Clinical Department, Bioness Neuromodulation, 43654 Ra'anana, Israel; ^3^Outpatient and Research Division, Reuth Medical Center, 67728 Tel Aviv, Israel; ^4^Department of Rehabilitation Medicine, Sackler Faculty of Medicine, Tel Aviv University, 69978 Tel Aviv, Israel; ^5^Biorobotics and Biomechanics Lab, Faculty of Mechanical Engineering, Technion-Israel Institute of Technology, 32000 Haifa, Israel

## Abstract

The study objective was to assess the effect of functional electrical stimulation (FES) applied to the peroneal nerve and thigh muscles on gait performance in subjects with hemiparesis. Participants were 45 subjects (age 57.8 ± 14.8 years) with hemiparesis (5.37 ± 5.43 years since diagnosis) demonstrating a foot-drop and impaired knee control. Thigh stimulation was applied either to the quadriceps or hamstrings muscles, depending on the dysfunction most affecting gait. Gait was assessed during a two-minute walk test with/without stimulation and with peroneal stimulation alone. A second assessment was conducted after six weeks of daily use. The addition of thigh muscles stimulation to peroneal stimulation significantly enhanced gait velocity measures at the initial and second evaluation. Gait symmetry was enhanced by the dual-channel stimulation only at the initial evaluation, and single-limb stance percentage only at the second assessment. For example, after six weeks, the two-minute gait speed with peroneal stimulation and with the dual channel was 0.66 ± 0.30 m/sec and 0.70 ± 0.31 m/sec, respectively (*P* < 0.0001). In conclusion, dual-channel FES may enhance gait performance in subjects with hemiparesis more than peroneal FES alone.

## 1. Introduction

A single-channel functional electrical stimulation (FES) system was first introduced fifty years ago by Liberson and colleagues to assist patients with hemiplegia demonstrating foot drop [1]. Since then, numerous studies have verified the benefits of peroneal stimulation for ameliorating foot drop and promoting motor recovery and locomotion [2–6]. These studies have demonstrated that peroneal FES significantly decreases fall incidence [4], increases walking speed [2–6], and improves gait rhythmicity and steadiness [4–6]. The results also suggest that the use of FES may potentially increase community participation and physical functioning [6]. Long-lasting therapeutic effects of FES for foot drop, which are maintained even when FES is not being delivered, have been demonstrated in the literature as well [2, 6].

However, many patients with hemiplegia and dorsiflexors inadequacy also demonstrate insufficient control of the knee flexors and extensors, which is essential for normal gait by providing shock absorption, assisting with foot clearance and balance control [7]. In fact, there is a moderate to strong significant relation between the strength of the knee extensors and flexors of the paretic limb and gait performance [8]. Consequently, FES to the thigh muscles may further enhance gait in patients with hemiparesis. Furthermore, as FES can be set individuality for each patient, it may address the variability in knee control deficits in patients with hemiparesis [9, 10]. For example, the quadriceps femoris muscle can be stimulated at the end of the swing phase to compensate for insufficient extension of the knee, or alternatively during midstance to provide greater confidence in shifting weight to the hemiparetic side. The hamstrings muscle can be stimulated at midstance to initial swing to decrease knee hyperextension and/or assist with leg clearance during swing.

Indeed, soon after the introduction of single-channel FES for foot-drop prevention, researchers started applying FES to muscle groups other than the foot dorsiflexors, with the muscles most often stimulated being the hamstrings and quadriceps muscles [11–16]. Although feasibility and some benefits of multichannel FES have been demonstrated, the few available studies vary to a great degree in terms of the stimulated muscle group, activation pattern, treatment length, and outcomes measured. More importantly, the research involving multichannel stimulation has focused mainly on evaluating the therapeutic effects of FES in patients at the initial stages of rehabilitation (acute phase) or in patients with severe motor disability, who are unable to walk independently [11–16]. However, many patients with chronic hemiplegia already living in the community still demonstrate gait disorders, often as a result of knee dysfunction. FES used as an active orthotic device to assist in controlling the ankle as well as the knee during gait may be beneficial in this population.

Therefore, the objective of this study was to investigate the effects of daily peroneal and thigh muscle FES on the temporal aspects of gait performance in individuals with hemiparesis who have walking ability, yet demonstrate ankle and knee dysfunction. We hypothesized that dual-channel stimulation will augment gait performance beyond the benefits of peroneal FES alone and that the immediate effects of dual channel stimulation will be further enhanced with a six-week habituation period.

## 2. Methods

### 2.1. Participants

Forty-eight subjects with hemiparesis were recruited for this study from outpatient clinics in rehabilitation centers in the central region of Israel. Inclusion criteria for subject selection were (1) diagnosis of an upper motor neuron lesion; (2) hamstrings or quadriceps strength of less than 4/5, as determined by manual muscle testing; (3) foot drop—toe drag during walking; (4) lower limb muscle tone 0–3 according to the modified Ashworth scale; (5) ability to walk independently or with an assistance device (e.g., cane, walker, etc.)/spot guarding for at least 10 meters; (6) ability to follow multiple-step directions, with a score greater than 21 on the Mini-Mental State Exam [17]; (7) sufficient response to electrical stimulation, that is, visible muscle contractions (at least 10° of movement) of each designated muscle (e.g., quadriceps, hamstrings, and tibialis anterior), as tested in a seated position. Exclusion criteria were a cardiac pacemaker, a skin lesion at the site of the stimulation electrodes, severe neglect (star cancellation test < 30), or major depression as defined using DSM-IV criteria.

### 2.2. The FES System

**Figure 1:**
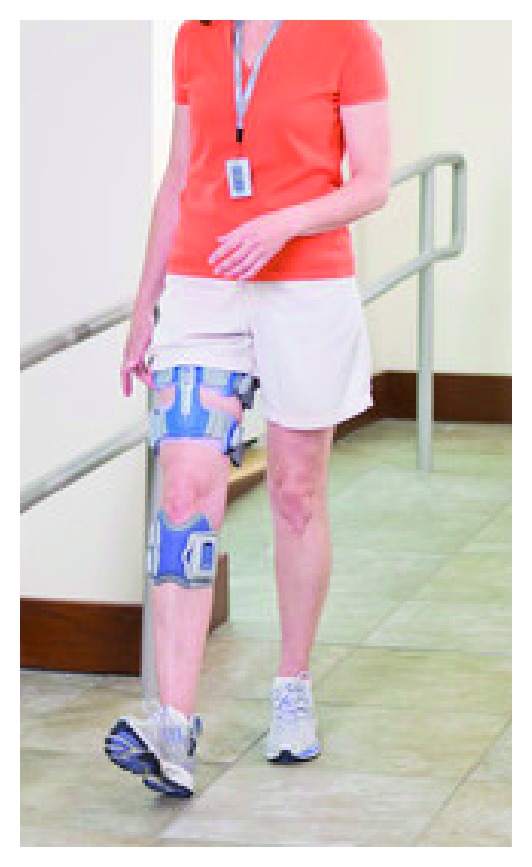
The NESS L300Plus.

The dual-channel FES system used in this study (NESS L300Plus, Bioness, Valencia, CA—Figure 1) delivers electrical pulses during gait to muscles in the affected leg in order to provide dorsiflexion of the foot and knee flexion or extension. The system consists of lower leg and thigh cuffs, a gait sensor, and a control unit that communicates by radio frequency signals. Each cuff integrates two electrodes and a stimulation unit. The electrodes of the lower leg cuff (two round 45 mm diameter cloth electrodes) are located over the common peroneal nerve and the tibialis anterior muscle to provide ankle dorsiflexion. The electrodes of the thigh cuff (two oval cloth electrodes, proximal: 130 × 75 mm; distal: 120 × 63 mm) are positioned over the quadriceps to extend the knee or over the hamstrings muscle to flex the knee. The gait sensor detects the force under the foot using a force-sensitive resistor. It uses a dynamic gait tracking algorithm analyzing the foot pressure to detect whether the foot is on the ground (e.g., initial contact) or in the air (e.g., heel off). Average stance and swing phases are calculated by the system and the data is transmitted by radio signals to the stimulation units allowing for the synchronization of the stimulation in accordance with the timing of gait events. A hand-held computer (PDA) is used by a clinician during the fitting process to set the stimulation parameters (e.g., intensity, pulse frequency) and the timing of the stimulation. To adjust the stimulation timing, stance and swing phases are represented to the clinician by the PDA's screen in a 5% resolution. The peroneal stimulation always starts when heel off is recognized and terminates with heel contact. In some patients, the clinician may extend the stimulation beyond heel contact to increase ankle stability. The duration of this “extended” period is defined by percentage of the stance period. The thigh stimulation (hamstrings or quadriceps) can start and end at any segment in the gait cycle, as defined by the clinician. Thus, for example, to assist with knee extension during terminal swing and knee stability during loading response, the clinician can set quadriceps stimulation from 85% of the stance period to 15% of the swing period. After the clinician sets the parameters, the patient is provided with a control unit which enables him/her to activate the system and receive information regarding its status (e.g., battery charging indications). The NESS L300Plus is based on the NESS L300 (for peroneal FES) and it utilizes the same gait detection algorithm.

### 2.3. Procedures

The study was approved by the Helsinki Ethical Committee of the Reuth Medical Center. All subjects signed an informed consent form prior to participation. Demographic and medical history data were obtained at the initial examination. All subjects were fitted with the dual-channel system, providing peroneal and thigh muscles FES. Stimulation parameters were initially set in a seated position until a visible movement of least 10° was observed (e.g., knee extension or flexion and ankle dorsiflexion). Stimulation intensity was readjusted during standing and walking to make sure that optimal movement was obtained (i.e., no under- or overcorrection). The predefined stimulation parameters set by the clinician were subsequently used by the subjects at home. In most cases, phase duration was 200 *μ*sec for peroneal stimulation and 300 *μ*sec for thigh stimulation; stimulation frequency was 30 Hz for peroneal stimulation and 40 Hz for thigh stimulation.

For those subjects who were already using the NESS L300 for peroneal FES, the system was upgraded to also include thigh stimulation (the NESS L300Plus).

In order to determine the appropriate location of the thigh cuff, two physical therapists independently assessed each patient's gait during a 10-meter walk at a comfortable pace, which was repeated twice. The thigh FES was applied to the muscle group that was most related to the observed knee dysfunction affecting gait. FES was applied to the quadriceps muscles in patients who demonstrated “knee crouch,” that is, increased knee flexion during the stance phase. In these cases, quadriceps stimulation was set from 0% to 80% of the stance phase. The system was applied to the hamstrings muscles in patients who demonstrated knee hyperextension during stance where hamstrings stimulation was set to 10%–90% of the stance phase or in patients with reduced knee flexion during swing phase where hamstrings stimulation was set from 80% of stance to 20% swing. In patients with excessive knee extension throughout most of the gait cycle (stiff knee gait pattern), the system was also applied to the hamstrings and was adjusted from 10% of stance to 20% of swing. When the physical therapists were not able to determine the most relevant muscle group for thigh FES, or the suitable timing of stimulation, several options were tested. The option offering the best correction was determined by visual inspection by two physical therapists. After fitting the dual-channel FES and adjusting the electrode placement and stimulation parameters in the leg and thigh cuffs, each patient underwent gait evaluations under three conditions introduced in a randomized order: with and without dual-channel FES, as well as with peroneal stimulation alone (only foot-drop correction with no stimulation of the thigh muscles). This initial assessment (T1) was followed by a six-week adaptation period, during which participants increased their daily use of the system according to a fixed protocol, so that by the end of the fourth week, all subjects were able to use the system for the entire day. A second identical assessment (T2) was conducted after this six-week period.

Under each walking condition in both assessments, temporal gait parameters were measured (i.e., velocity, gait asymmetry, and single-limb stance percentage) during a two-minute walk test (2MWT), using force-sensitive insoles placed in the subjects' shoes (B&L Footswitches, Tustin, CA), which were connected to a data logger (JAS Research Inc., Belmont, MA) [4–6]. Under each 2MWT condition, the subjects were instructed to walk as far as they could, at their self-selected normal walking speed, back and forth along a 50-meter hallway, turning around each time they reached the end of the walkway. The distance walked in two minutes has been shown to correlate well with the longer 6- and 12-minute walk tests [18] and was selected to minimize fatigue effects.

Average gait speed was determined by dividing the distance covered in two minutes by 120 seconds. The gait asymmetry index was measured and calculated as a marker of interlimb coordination, as follows: 100 × {(swing time paretic − swing time nonparetic)/(swing time paretic + swing time nonparetic)}. When the swing asymmetry index = 0, gait is perfectly symmetrical, while higher scores indicate a lack of symmetry, a measure that has been associated with poor balance and a high risk for falls [4, 19]. The percentage of a single-stance phase was calculated as the percentage of time in the gait cycle spent as single stance on the paretic limb (equal to the swing phase of the nonparetic limb). To imitate daily life situations, average gait speed was also determined by measuring the time spent to walk 10 m over an obstacle course, using the protocol in the Emory Functional Ambulation Profile [20]. Finally, a feedback questionnaire was filled out by the subjects at the end of the study period in order to evaluate their perceptions regarding the usability of the FES system.

### 2.4. Statistical Analysis

Four gait parameters were defined: (1) two-minute gait velocity, (2) obstacle course gait velocity, (3) the asymmetry index, and (4) the percentage of a single-stance phase on the paretic limb. Descriptive statistics included means and standard deviations (SD) for numerical variables and frequencies for categorical variables. Due to the lack of normal distribution, nonparametric analysis was used. Friedman's test was used to compare results of the three gait conditions (i.e., no stimulation, peroneal FES alone, and peroneal and thigh FES) at baseline (T1) and after six weeks (T2). Post hoc analysis comparing all pairs of conditions (separately at T1 and T2) was performed using Holm's method for multiple comparisons. Wilcoxon's matched pairs test was used to compare between the performances at T1 and T2 during the combined peroneal and thigh FES. Significance was determined at *P* < 0.05. For Friedman's and Wilcoxon's matched pairs tests, *P* values of < 0.0125 (0.05/4) were considered as significant after applying the Bonferroni correction. For the post hoc analysis, critical values were determined according to Holm's method.

## 3. Results

### 3.1. Subjects Characteristics

Of the 48 subjects who were recruited to the study, three subjects withdrew consent after one week due to their inability to attend follow-up visits. The results refer to the analysis of the 45 patients who completed the study. The average age of the patients was 57.8 ± 14.8 years, and 29 patients (64%) were male. Forty patients (89%) were after CVA, while the remaining patients were diagnosed with a brain lesion due to resection of a brain tumor (*n* = 3) or a traumatic brain injury (*n* = 2). The average time since diagnosis was 5.37 ± 5.43 years, and 24 patients (53%) had right-side hemiparesis. Five patients were in the subacute phase of rehabilitation (i.e., 2–6 months since the insult), while 40 of the subjects (89%) had chronic hemiparesis (i.e., more than six months since the insult). Prior to initiation of the study, 38 of the subjects (84%) had used a device to correct their foot drop, with 23 patients (51%) using the NESS L300, 13 using an ankle-foot orthosis (29%), and 2 (4%) using a Dictus band foot-drop aid (Erimed International KB, Sweden). 

### 3.2. Gait Performance

All patients were able to walk with the dual channel FES immediately after fitting, with 39 patients (87%) using the system with the thigh FES applied on the hamstrings and six patients (13%) with it applied on the quadriceps.  Table 1 summarizes the group means and standard deviations of all measured temporal gait outcomes at each assessment (T1—study initiation; T2—after six weeks of daily use) and presents the Freidman's test analysis results. As the table shows, significant condition effects were found at each assessment. 

Table 2 shows the Holm's post hoc analysis of multiple comparisons. The comparison between the peroneal stimulation and no stimulation conditions demonstrated a significant orthotic effect in all variables, with the exception of single-limb stance percentage at T1. It should be noted that the effect of the peroneal and thigh stimulation in comparison to no stimulation was significant for all variables.

**Figure 2:**
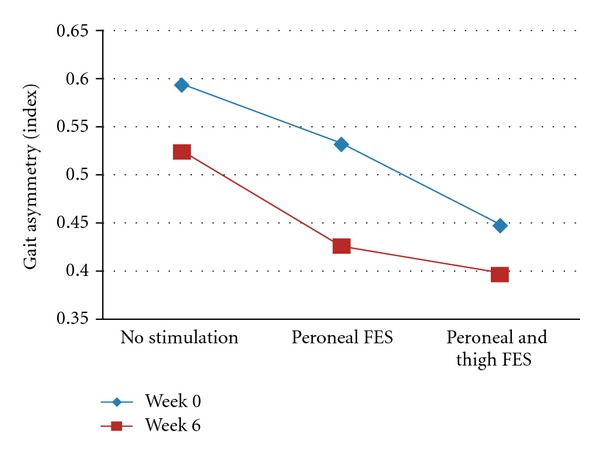
Effects of FES on gait asymmetry.

**Figure 3:**
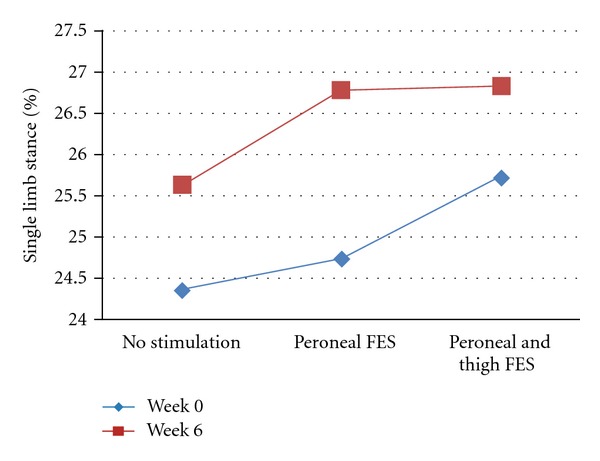
Effects of FES on single-limb stance percentage.

The post hoc tests comparing performance between peroneal and thigh FES and peroneal FES alone indicated further significant improvement with the dual-channel FES. Gait velocity (measured by two-minute gait speed and obstacle course gait velocity) was enhanced with peroneal and thigh FES, as compared to peroneal stimulation alone, at both assessments. For example, at T2, the two-minute gait speed measurements with peroneal FES alone and with the dual channel FES were 0.66 ± 0.30 m/sec and 0.70 ± 0.31, respectively (*P* < 0.0001), and the obstacle course gait velocity measurements were 0.40 ± 0.20 m/sec and 0.43 ± 0.21 m/sec, respectively (*P* < 0.0001). Additional significant differences in gait dynamics were demonstrated in the comparison of the two FES conditions. At T1, the single-limb stance with the dual-channel FES (25.74 ± 6.34%) increased in comparison to that of peroneal FES alone (24.75 ± 7.32%, *P* = 0.005) (see Figure 2). At T2, the gait asymmetry index, a marker of gait stability, improved by 8% from 0.43 ± 0.26 to 0.40 ± 0.30 (*P* = 0.006) (see Figure 3). 

Wilcoxon's matched pairs test comparing performance with dual-channel FES at T1 and T2 showed significant improvements at T2 in both outcomes of gait velocity (two-minute gait speed and obstacle course gait velocity; *P* < 0.0001 for both measures). A clear trend of training effect was found for the single-limb stance percentage (*P* = 0.018; after applying Bonferroni correction significance was set to *P* = 0.0125), while the gait asymmetry did not change (*P* = 0.132).

### 3.3. User Acceptance

Table 3 summarizes the subjects' perceptions regarding usability of the peroneal and thigh FES system. All patients described the system as safe for use, and 87% were enthusiastic about continuing to use it. Furthermore, 93% of the subjects described their walking ability as better or significantly better while using the dual-channel system; 78% reported more confidence in walking; 84% felt greater confidence in walking on inclines and/or uneven ground while using the system. As can be seen in Table 3, the majority of the subjects were satisfied with the ease of adjusting the system (84%), reporting that it was not difficult to place the cuff in the correct position on the thigh and leg and that they rarely needed assistance with operating the system after the initial training (69%). Moreover, most of the participants (76%) found the system to be comfortable for all-day use and that it allowed them to perform more daily tasks and activities. Eleven patients reported a mild skin abrasion under the cuff, but all such cases were resolved after readjustment of the cuff's straps. Skin irritation under the stimulation electrodes was neither reported nor observed. 

## 4. Discussion

The study's findings suggest that dual-channel FES, applied daily to the peroneal nerve and thigh muscle by individuals with hemiparesis, positively affects gait performance. The beneficial effects of this dual-channel application were superior to those obtained by peroneal stimulation alone, which is the most common application of FES in this population. To our knowledge, the present study is the first to document these advantages.

Significant advantages were demonstrated when comparing the gait speed achieved with the dual-channel FES and those achieved with peroneal stimulation alone (Tables 1 and 2). It is well accepted that improving gait speed is an important rehabilitation goal [21] and that increased gait velocity is associated with better function and quality of life [6]. After the conditioning period, the dual-channel FES significantly improved the participants' gait speed by a mean of 0.04 m/sec in comparison to peroneal stimulation alone. Although the differences were relatively small, previous research findings indicate that even such small improvements in gait speed are sufficient to detect real clinical changes in patients following a stroke [22]. Thus, for example, Perera et al. [23] estimated a change of 0.04–0.06 m/sec in gait speed as a small meaningful change. 

In addition to the changes in gait velocity, after six weeks, the gait asymmetry index was improved with peroneal and thigh FES as compared to peroneal FES alone. Gait asymmetry is a measure of interlimb coordination, which is not necessarily related to gait velocity, but provides insight regarding the underlying mechanisms that control gait [19, 24, 25]. Additionally, gait asymmetry is a measure that is not easily modified by conventional physical rehabilitation approaches [5, 26].

In contrast to gait asymmetry, the other measure of gait dynamics, namely, the single-limb stance percentage, was not significantly different between the two FES conditions at T2 (after six weeks). This may be due to the enhanced results that were achieved with peroneal FES alone at the six-week test. While at the beginning of the study a comparison of the no stimulation versus peroneal FES conditions did not yield significant results, such a comparison was found to be significant after six weeks (see Figure 3).

Our protocol included two points of evaluation (i.e., immediately after fitting and after six weeks of adaptation). In studies involving foot-drop stimulation, it has been shown that a period of four to eight weeks is necessary to achieve an optimal orthotic effect [4, 27]. Our findings are consistent with previous studies by showing improved orthotic effect with the peroneal and thigh FES after the adaptation period. The two-minute gait speed and obstacle course gait velocity were enhanced at T2 and a clear trend of training effect was found for the single-limb stance percentage. It is possible that longer use with the dual-channel system may result in additional gains in gait performance, as demonstrated in previous studies with peroneal FES [6].

Many factors may have contributed to the results presented in this study. Given that walking speed has been shown to be positively correlated with knee flexion during swing [28], it is possible that the enhanced gait speed observed in our study resulted from improved knee flexion. The hamstrings/quadriceps FES during stance may have provided the patients with greater confidence in shifting weight to the hemiparetic side, leading to a more symmetrical gait. An additional factor that may have contributed to the results is the ability to tailor the temporal parameters of the electrical stimulation according to the needs of the individual subject.

Intriguingly, although thigh stimulation was applied according to the knee dysfunction most prominent affecting gait, only 13% of the patients used the system for quadriceps muscle stimulation. This uneven distribution may be explained by the classic pattern of central lesions that affect flexors more than extensors in the lower limb, also known as the “pyramidal” pattern [29]. Another possible explanation may be attributed to the inclusion criteria in this study. Patients included in the study had to have the ability to walk independently or with an assistance device/spot guarding for at least 10 meters. Consequently, they may have had sufficient quadriceps strength, with a lower tendency for “knee crouch,” in order to meet this demand.

The daily use of a multichannel FES is not trivial. Difficulties may arise in relation to factors such as electrodes positioning, user interface, and overall convenience. Yet, the participants' feedback about the device used in the present study was very positive, with no major difficulties reported in regard to operating the system or placing the stimulation cuffs. The majority of the subjects rated the dual-channel system as the most useful system for assisting in their gait and function (78%) and were enthusiastic about continuing to use it (87%). Despite the benefits, however, the use of this device has several restrictions. The location of the thigh cuff under the clothing may be cumbersome for some patients. In addition, skin irritation might develop after prolonged use with electrical stimulation.

This study has several limitations, including the protocol duration of six weeks, the lack of a control group, and the nonnormal distribution of the results. Further investigations should be undertaken to confirm the study results with appropriate control groups and longer durations of use. It is also possible that a larger and more homogenous sample in terms of gait ability prior to the intervention would have resulted in more normally distributed results enabling a more comprehensive analysis. Finally, kinetic and kinematic studies may be useful in understanding the underlying mechanism of the effects of dual-channel FES. The promising results of the present investigation suggest that such studies are warranted.

## 5. Conclusions

Functional electrical stimulation is an accepted treatment method for paresis or paralysis after stroke, as well as for other neurological upper motor neuron disorders. In the past, implementation of FES focused primarily on the stimulation of ankle musculature. The findings of this study suggest that the application of FES which is intended to assist with ankle and knee activation may further improve the temporal characteristics of gait in patients who demonstrate insufficient knee control in addition to foot-drop. Thus, the results of this study may lead to a more effective application of FES technology.

## Figures and Tables

**Table 1:** Group means, standard deviations of all measured gait performance variables, and results of analysis of Freidman's test.

	Outcome measure	No stimulation	Peroneal stimulation	Peroneal and thigh stimulation	Freidman's test
T1	2 min walk test (m/sec)	0.50 ± 0.25	0.55 ± 0.26	0.58 ± 0.28	<0.0001
	Obstacle walk (m/sec)*	0.28 ± 0.19	0.35 ± 0.19	0.37 ± 0.20	<0.0001
	Asymmetry index	0.59 ± 0.42	0.53 ± 0.52	0.45 ± 0.27	0.0004
	Single stance (%)	24.37 ± 6.68	24.75 ± 7.32	25.74 ± 6.34	0.0030

T2	2 min walk test (m/sec)	0.58 ± 0.29	0.66 ± 0.30	0.70 ± 0.31	<0.0001
	Obstacle walk (m/sec)*	0.33 ± 0.20	0.40 ± 0.20	0.43 ± 0.21	<0.0001
	Asymmetry index	0.52 ± 0.33	0.43 ± 0.26	0.40 ± 0.29	<0.0001
	Single stance (%)	25.64 ± 6.17	26.78 ± 5.98	26.83 ± 6.41	<0.0001

*Obstacle test that could not be finalized because of difficulty was assigned the value 0. Following are the number of patients out of a total of 45 who completed the test: at T1: no stimulation—*n* = 37; peroneal stimulation/peroneal and thigh stimulation—*n* = 43; T2: no stimulation—*n* = 41; peroneal stimulation/peroneal and thigh stimulation—*n* = 45.

**Table 2:** Post hoc analysis comparing all pairs of conditions (at T1 and T2). *P* values are presented only when the results were significant (*P* value < Holm's critical value).

	Outcome measure	No stim. versus peroneal stim.	No stim. versus peroneal and thigh stim.	Peroneal versus peroneal and thigh stim.
T1	2 min walk test (m/sec)	<0.0001	<0.0001	<0.0001
	Obstacle walk (m/sec)	<0.0001	<0.0001	0.0003
	Asymmetry index	0.0003	0.0002	NS
	Single stance (%)	NS	0.0004	0.0051

T2	2 min walk test (m/sec)	<0.0001	<0.0001	<0.0001
	Obstacle walk (m/sec)	<0.0001	<0.0001	<0.0001
	Asymmetry index	<0.0001	<0.0001	0.0062
	Single stance (%)	<0.0001	<0.0001	NS

Stim. = stimulation.

**Table 3:** Summary of the subject's acceptance questionnaire.

Question	Answer and frequency (%)		
How do you feel about continuing to use the L300Plus?	Unenthusiastic 3 (6.5%)	Indifferent 3 (6.5%)	Enthusiastic 39 (87%)
How would you rate the L300Plus against other aids to assist your gait and function?	Less useful 1 (2%)	As useful 9 (20%)	More useful 35 (78%)
How much help did you need in operating the L300Plus?	I needed assistance almost each time 3 (6.5%)	I occasionally needed assistance 11 (24.5%)	I rarely needed assistance 31 (69%)
How satisfied are you with the dimensions (size, height, length, width) of the L300Plus?	Not satisfied 7 (16%)	More or less satisfied 17 (37.5%)	Satisfied 21 (46.5%)
How satisfied are you with the ease in adjusting (e.g., donning and doffing) the L300Plus?	Not satisfied 7 (16%)	More or less satisfied 19 (42%)	Satisfied 19 (42%)
How would you describe using the L300Plus during the day?	Inconvenient 11 (24.5%)	Convenient 28 (62.5%)	Very convenient 6 (13%)
How would you describe your walking ability while using the L300Plus?	Same 3 (6.5%)	Better 22 (49%)	Significantly better 20 (44.5%)
While using the L300Plus, has there been a change in your ability to perform daily tasks activities?	Can perform fewer activities 3 (6.5%)	Can perform the same 8 (18%)	Can perform more activities 34 (75.5%)
How would you rate your confidence in walking with the L300Plus system versus without it?	Less confident 0 (0%)	No difference 10 (22%)	More confident 35 (78%)
Do you feel greater confidence in walking on inclines and/or uneven ground while using the L300Plus?	No 7 (16%)	Yes 38 (84%)	
Do you find the use of the L300Plus safe?	No 0 (0%)	Yes 45 (100%)	
Would you recommend a person with your condition to use the L300Plus?	No 2 (4%)	Yes 43 (96%)	
